# Case of a Sister Mary Joseph's Nodule in a High Grade Serous Carcinoma

**DOI:** 10.7759/cureus.3757

**Published:** 2018-12-20

**Authors:** Matthew T Carvey, Cristina V Beiu, Robert Hage

**Affiliations:** 1 Internal Medicine, St. George’s University School of Medicine, St. Georges, GRD; 2 Dermatology, Elias Emergency University Hospital, Bucharest, ROU; 3 Otolaryngology, St. George's University School of Medicine, St George's, GRD

**Keywords:** umbilical nodule, metastatic cancer, intra-abdominal cancer, gynaecological cancer, sister mary joseph nodule

## Abstract

Sister Mary Joseph's nodule (SMJN) is a metastatic malignancy of the umbilicus usually indicative of advanced, metastatic disease. It is a rare occurrence, but it may be the first sign of abdominal cancer, most commonly an adenocarcinoma metastasis from a gastrointestinal or gynecologic primary malignancy. We present the case of an 82-year-old woman with an acute, 2 cm non-tender mass located at the umbilicus, diagnostic indication of an SMJN. Additional investigations included imaging via ultrasound and a pelvic magnetic resonance imaging (MRI) along with a biopsy and immunohistochemical staining in order to further characterize the mass. Our findings are discussed in the following case report.

## Introduction

Sister Mary Joseph's nodule (SMJN) is a rare growth found at the umbilicus that indicates a metastasis from a pelvic or abdominal source. Cutaneous metastases of malignant neoplasms are relatively uncommon, occurring between 0.7% and 9% of autopsy evaluations [1]. An SMJN typically presents as an umbilical nodule less than 5 cm in diameter, but nodules may be up to 10 cm, and commonly express bloody and purulent discharge. After ruling out the benign differential diagnoses of an umbilical nodule, the concern of metastasis arises. Metastases can occur via hematogenous or lymphatic routes, contiguous extension, embryologic remnants, ventral hernias, or be iatrogenic in nature [2]. The presence of an SMJN, in conjunction with other clinical symptoms such as weight loss and epigastric tenderness, can be suggestive of intra-abdominal or gynecologic cancers that have metastasized to the umbilicus. The evaluation of an umbilical mass should be directed by suspicion of a metastatic deposit, keeping in mind its potential to be either a primary malignant umbilical lesion or a benign disease [3]. In women, the most common origin of SMJN is ovarian carcinoma, while in men, it is a gastric carcinoma [4]. Recognition of the nodule is of great importance because it may be the first presenting sign in a patient with a previously unknown malignant disease [5]. While umbilical hernias and foreign bodies are far more common pathologies of umbilical nodules, the literature is replete with cases masquerading as umbilical hernias that ultimately are determined to be metastatic deposits [6]. The aim of this case report is to emphasize the importance of correctly diagnosing an umbilical nodule in order to accurately evaluate the possibility of malignancy.

## Case presentation

**Figure 1:**
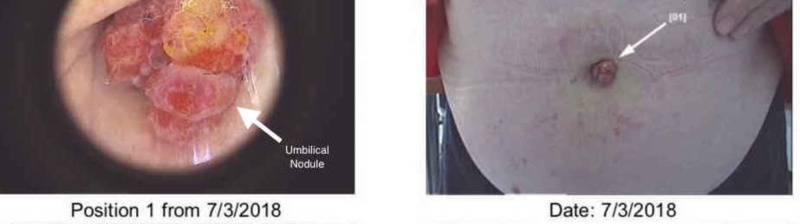
Dermascopic and macroscopic view of a protrusive, bloody, purulent nodule of the umbilicus The mass measured 2 cm in diameter.

An 82-year-old obese woman presented to the dermatology department because of a two-month history of an enlarging umbilical mass that had been bleeding. The patient also complained of menorrhagia for the previous two weeks. Physical examination showed a 2 cm firm, non-tender, protrusive umbilical nodule (Figure 1). Laboratory studies showed moderate anemia and a high human epididymis protein 4 (HE4) marker. The Risk of Ovarian Malignancy Algorithm, or ROMA score, classified this patient at high risk for malignant disease. An abdominopelvic ultrasound examination showed a right ovarian mass and a right parauterine teratoma. A solid hypo-echoic mass in the umbilicus without any sonographic features of inflammation involving the adjacent soft fatty tissue was suggestive of an SMJN, and led to a search for the primary tumour and other metastases [7].

Histological and immunohistochemical examination of the umbilical mass biopsy diagnosed a high-grade serous ovarian carcinoma. Histological/cytological evaluations of all umbilical lesions are mandatory, not only to determine its nature but also to guide the clinician in searching for the possible primary source [8]. Further assessment with MRI confirmed the diagnosis and detected a lymph node metastasis in the right external iliac group.

The patient subsequently underwent a hysterectomy and bilateral salpingo-oophorectomy. The patient declined chemotherapy as she found this treatment approach too aggressive.

## Discussion

It is suggested that primary umbilical adenocarcinomas arise from embryologic remnants connected to the umbilicus, such as the omphalomesenteric (OM) duct or the urachus [9-10]. The OM remnant is the embryological connection between the yolk sac and midgut lumen. If the OM persists in adulthood, it can lead to clinical findings of a Meckel’s diverticulum, vitelline fistula or become a connection between the gastrointestinal system and umbilicus from which metastases, such as in the SMJN, can arise. The metastatic infiltration of the umbilicus is thought to be by direct invasion of the peritoneum, through lymphatic or blood vessels, or via embryonic remnants [11].

Diagnosis of SMJN was made. Consideration of the primary differential diagnoses of a patient presenting with an umbilical nodule included an umbilical hernia, endometriosis and a keloid, with other causes including a granuloma, abscess, mycosis, and eczema (Table 1) [12]. All included differential diagnoses were considered and ruled out on further investigation into each.

**Table 1:** Summary of the differential diagnoses and treatment options related to Sister Mary Joseph's nodule (SMJN)

Differential Diagnosis	Treatment Options
Umbilical Hernia	Open or laparoscopic repair
Endometriosis	Analgesia and hormone therapy
Keloid	Corticosteroids and pressure earrings
Granuloma	Corticosteroids
Abscess	Drainage and antibiotics
Mycosis	Antifungal agent
Eczema	Topical corticosteroids

An umbilical hernia occurs when the abdominal musculature fails to close completely in infancy, or excess pressure to the abdominal contents is present, resulting in penetration of intestinal tissue at this location. Adult umbilical hernias usually are a consequence of increases in pressure (e.g., pregnancy or ascites) combined with the pull of the abdominal muscles and the deterioration of the connective tissue [13]. An umbilical hernia can be ruled out via an abdominal ultrasound or computed tomography (CT) scan. 

Endometriosis results when tissue normally found in the uterine-lining is found outside of the uterus. Umbilical endometriosis is a common manifestation of external endometriosis, representing primary or secondary endometriosis, with a typical presentation that has little variation [14]. This differential was ruled out based on patient presentation and histological analysis of the mass showing non-inflammatory features.

A keloid is cell growth beyond the boundaries of normal cell division. The exuberant scar tissue in keloids has been attributed to augmented growth factor activity (transforming growth factor-β and platelet-derived growth factor) and alterations in the extracellular matrix (fibronectin, hyaluronic acid, and biglycan) [15]. A keloid was ruled out in this case based on the histological results obtained, showing an absence of such proliferation.

Treatment strategies for an SMJN focus on diagnosing the underlying cause of the metastatic tissue. Once discovered, recent studies recommend an aggressive surgical approach combined with chemotherapy as a treatment strategy for an SMJN associated metastasis [16]. Yet as the SMJN is often a sign of advanced malignancy, this treatment rarely improves survival [17]. SMJN has traditionally been considered a sign of ominous prognosis (survival of 10 months on average) [18].

## Conclusions

Umbilical masses in adults are rarely seen in day-to-day practice, and physicians must exercise caution when dealing with umbilical lesions. As in this patient’s case, an SMJN can be an important diagnostic and prognostic factor to consider in the assessment of gynecologic oncology patients. Therefore, these nodules should always prompt further investigation into the primary cause of the growth.
